# History of Two Cases of Burn Producing Serious Constitutional Irritation

**Published:** 1830

**Authors:** Daniel Drake


ART. VIII.—*History of two Cases of Burn, producing serious constitutional irritation.* By DANIEL DRAKE, M. D.

The readers of the Western Journal will recollect, that in the Autumn of 1828, its publication was suspended, in consequence of a burn which the Editor received on both hands in extricating one of his female relatives from the flames. The object of this paper is to report such facts connected with that casualty, as may be interesting to the profession.

*Case* 1. A young lady, aged 20 years, in feeble health, was extensively burned, by her musquitoe curtains and calico bed cover being set on fire, after she had fallen asleep. The flames enveloped her head, neck and arms, and were not extinguished for 20 or 30 seconds. Ardent spirits were first applied, then flaxseed oil, and, lastly, a calcarious soap of the same oil and lime water. Laudanum was administered freely, both externally and internally.

The pain and smarting were intense. Great coldness soon came on, with a weak pulse, and epigastric anxiety. Her mental faculties continued unimpaired. In a short time her respiration became irregular, and stertorous. Coma supervened, and about 8 hours after the accident she expired. No reaction took place, and she died from the shock which her nervous system suffered. As her face was surrounded by the flames, and her mouth burnt, it is not improbable they were drawn into the fauces, even as far as the glottis.

*Case* 2. The Editor, in rescuing the patient, whose case has just been related, was severely burnt in both hands. It happened by compressing the burning clothes in his naked hands, until the flames were extinguished. The cuticle exfoliated from nearly the whole of the right hand, and the cutis vera sloughed in seven places, of which the deepest was the ball of the middle finger. The left hand suffered much worse. The whole cuticle was separated, and much of it was rolled up, especially that which covered the space between the wrist and hollow of the hand, extending up the inner side of the thumb, as far as the middle of the second phalanx. Between these limits, and the metacarpal bone of the little finger on one side, and the corresponding bone of the thumb on the other, the true skin, was killed and turned black. The back of the wrist and hand suffered almost in an equal degree, and the ball of the forefinger was still more deeply injured.

Immediately after the accident he immersed both hands in whiskey, then in a liniment of flax seed oil and lime water, after which the affected parts were wrapped in rags dipped in a painter's mixture of the same kind of oil and white lead.

ground together. In three days this was replaced by carrot poultices with yeast and peruvian bark ; the deeply burnt, parts being stimulated at different times with tincture of myrrh, oil of turpentine, ammoniated alcohol, and other excitants. Elm bark poultices were then used, and subsequently the cerate of lapis calaminaris, and yellow resinous ointment. After a few days, suppuration was established and the dead skin sloughed off. In the ordinary time granulations arose, and at the end of six weeks an imperfect cicatrization had taken place. Thus the cure of the burnt parts, considering their depth and extent, was effected in good time, by the usual means, and had the case presented nothing but what has been stated, a report of it would have been of little interest.

There resulted, however, from this local injury a great constitutional disturbance. And to this I beg leave to direct the attention of the reader.

The temperament of the patient was the bilious slightly modified by the lymphatic. Of course so grave an accident was more likely to be followed by a state of nervous, than vascular irritation ; and this, in its most distressing form, actually supervened. Several causes seem to have conspired to depress the energy and disorder the sensibilities of his nervous system.

1. The acute sensibility of the parts injured, resulting as in the case of medical men generally, from the use of the hands in a great variety of delicate manipulations. In consequence of this state of exalted sensibility, the pain, for several hours after the accident, was excruciating, notwithstanding the liberal use of laudanum.

2. The scene of concentrated horror, under which the injury was inflicted, contributed not a little to overthrow the powers of the nervous system.

3. The immersion of both hands, extensively denuded of cuticle, in a liniment of oil and white lead, seems to me to have contributed largely to the same effect. The direct influence of the preparations of lead on the nerves of the part to which they are applied, and through them, upon the system at large, is unquestionable. To this power we may refer the effects which flow from solutions and liniments of the acetate of lead in external inflammations, which are well known to diminish the energy of the nervous function of the part, and, consequently, to subdue the inflammation more effectually than cold or evaporating washes of simple water.

At a time, and in a country where the paste of white lead ground in oil, is a fashionable application to burns, I feel disposed to exhort those who may employ it, to be cautious as to the extent of surface over which it may be spread, and the length of time it may be left on the injured skin.

In the case now under consideration, not only an augmented nervous irritation was the consequence of such an application; but likewise *a real saturnine colic*—consisting in obstinate constipation, retraction of the umbilicus, borborigmi and acute abdominal pain, which came on about four days after the white lead was applied. On mentioning this fact to a medical friend, he informed me, that 18 months previously, he saw a case of burn treated with the same application, and the patient during his convalescence was afflicted with severe colics, to which, neither before nor since, had he been in any degree liable, and for which there was no other obvious cause than the white lead.

4. Two, three or four days after the accident, when febrile symptoms were developed, the patient, by his own advice lost about 10 ounces of blood. This was done under the apprehension that the fever and inflammation might run too high, for healthy suppuration, but it manifestly did harm.

It would be difficult to set forth the variety of physical and moral sufferings, which were attendant on the protracted state of nervous or constitutional irritation which these causes generated.

Much of the time the pulse was not marked by any *great* peculiarity, being near the healthy standard in frequency, though below it in force and fullness, but occasionally it was frequent and feeble. The secretory functions, generally, were deeply smitten and carried on most imperfectly. That of the kidneys* was much diminished, and for several days a true ischuria existed, so that the moderate quantity of urine secreted was retained in the bladder until that organ was stimulated into action by medicinal agents. Of these, the application of heat and moisture to the hypogastric region, emollient enemata, the tinctura muriatis ferri and a mucilage of elm-bark (Ulmus Americana. Cortex) were the most successful.* The secretion of bile was suspended in a still higher degree, and could not be restored by mercurials even in large doses. Nothing but a nourishing and savoury diet, at the end of several weeks effected this object. In this there was not any thing which ought to be considered remarkable ; for food is in fact the natural stimulus of the liver, and I have more than once seen it effect the restoration of the biliary secretion after other means had failed. When patients not of a sanguine temperament, are subjected to a very rigid abstinence, the secretion of bile is apt to fail, and the suspension is too often regarded as a cause instead of an effect, a fact which I have known to be overlooked to the detriment of the patient.

The appetite was not greatly impaired but fastidious, and the contents of the stomach were sometimes thrown up. The bowels were torpid and much contracted. The secretions of the mucous membrane were evidently reduced in quantity, and the powers of evacuation diminished. For some time the rectum was almost paralytic, and so insensible, that enemata of the most stimulating kind were scarcely felt by the patient. Ten or twelve days after the accident, a hæmorrhoidal tumor formed at the extremity of the bowels, and was endowed with the intense morbid sensibility. The patient had been liable to such attacks for many years, and it is worthy of remark, that until

*I take this opportunity to inform the profession, that Mr. Christopher Carey, an inhabitant of the Miami country, has invented a method of pulverizing this bark, so that it readily dissolves in boiling water—forming either an elegant mucilage for internal nse, or a most soothing and useful cataplasm. For these purposes it is now kept in the principal druggists' shops of this city, and is worthy of a place on the shelves of every physician and surgeon. It should be kept in close vessels, as some kind of insect deposits its eggs in the powder when exposed, and small hairy larvæ are hatched out.

18 months after his recovery he did not experience the slightest return of that malady, and then it was reproduced by an aloetic pill.

The cutaneous functions were equally impaired with the chylopoietic. The skin became and remained pale and bloodless, perspiration was nearly checked, and the power of generating heat greatly reduced.

These morbid states of the secretory organs, were sufficiently indicative of an overthrow of the functions of that system, which presides over their various operations ; but the feelings of the patient were still more significant of nervous depression and perversion. A sense of muscular debility and a consciousness of mental imbecility, extreme restlessness, and morbid vigilance, were the utterable sensations which can be recollected. None of the ordinary stimuli of life, either moral or physical, produced their characteristic effects, the system being insensible to their presence, or the effects which they produced being different from those which they excite in health. In short every sensation both of mind and body, was unpleasant if not painful ; and this continued to be the case for at least five weeks.

For several years the patient had been subject, with but little interruption, to daily paroxysms, chiefly in the afternoon, of cerebral oppression accompanied with drowsiness, suffusion of the face, and a sense of sinking and vacuity *in epigastrio.* These symptoms had come on both before and after venesection, and would fairly have been regarded by Dr. M'Cullough, as an example of chronic and relapsing intermittent. With this previous tendency to a diurnal paroxysm, the constitutional irritation which I have just described, was soon observed to coincide, and for a number of days, the patient had an afternoon exacerbation, which might have passed for a fit of what is denominated congestive intermittent fever :—The temperature, force of the circulation, and activity of the nervous system, being so reduced, as, apparently, to threaten dissolution. The hour at which this paroxysm recurred was observed to vary for some time, becoming later and later as frequently happens with intermittents.

It deserves to be mentioned, that the process of granulation in the burnt parts advanced in a favourable and healthy manner in the midst of this extreme constitutional irritation.

It is, also, worthy of being stated, that for a twelve month or more after the accident, the cerebral affection was nearly suspended, but has since that time been gradually returning, though it is much less severe than before the burn.

For the cure of this disorder, a great variety of means were employed with but small effect. An emetic which so often revives and regulates the sensibilities of the system, afforded but little relief. Tonics, embracing the sulphate of quina in large doses, were beneficial, but their powers were inadequate to a cure. During the afternoon paroxysms, when the temperature and vital forces were greatly reduced, external and internal stimulation of the most energetic kind was employed, and seemed *immediately* necessary to the preservation of life. Sulphuric ether and ammoniated alcohol were serviceable ; but opium and the preparations of morphia, were found most efficacious ; and could not be dispensed with every evening for more than thirty five. I cannot too strongly recommend the sulphate or acetate of morphia to all who labor under constitutional irritation, with painful local affections. When it cannot remove the latter, it possesses the power of *reconciling* the patient to them, an important *desideratum.* Piperine was taken for two days, but its effects were much less, than those of morphia. The patient made an observation, however, which for its physiological importance deserves to be recorded. After taking the medicine for 24 hours in the form of pills, he could taste it in his saliva, or at least had constantly present in his mouth a piperine or peppery taste, not referable to any contact of the medicine with that part. Exercise in the open air, was of course regarded as more likely to prove efficacious than any other means ; and, accordingly, the patient was carried abroad even before he was able to sit erect. "An airing" was found, however, to do but little good. His senses were unsusceptible to external impressions, and when surrounded by the objects and scenes which in health impart pleasure both to the body and mind, he could elicit from them no gratification to either He recognized their presence, but felt not their animating influence, although it was anxiously desired. Still, however, on principle, it was clear, that exercise ought to break the spell which bound his physical system, and it finally did. Being placed on board a steam boat, destined for Louisville, the incessent gestatory motion, in a few hours, began to revive the pleasurable feelings of health, and he was enabled to pass the night of the voyage, without an opiate—the first for more than a month. He slept but little, yet he escaped the usual paroxysm of restlessness and prostration. The next day he was enabled to endure a carriage ride ; the following day to relish it ; and on the third to *enjoy* the return voyage with disenthralled sensibilities. From that time forward, his recovery was regular and rapid. Though a brief evening paroxysm of nervous irritation occasionally required a diffusible stimulus.

I have been the more prolix in these details, because those states of chronic nervous irritation which no medicine can cure, are not uncommon ; may spring from various causes, afflict persons of both sexes and of different ages ; subject the unfortunate patient to the derision of his acquaintances, and too often bring upon his constitution the desolating tortures of officious medication. I have seen many persons in this sad and pitiless state of physical and moral imbecility, and can bear testimony to the great uncertainty of medical prescriptions for their relief ; indeed I have often known them augment the very irritation they were expected to subdue ; and am compelled to believe, that many of the nervous diseases which are so often prolonged through an indefinite period, are kept up by our *polypharmacia antispasmodica.*

In these cases, so distressing to the patient and so perplexing to the physician, the best resource is exercise in the open air, with an abandonment of all active and irritating medicines. In many instances the plan will be of difficult execution, for the patient will be alarmed at the very suggestion of exercise ; but it soon recommends itself by its beneficial effects, and if no organ is permanently deranged in structure, it will, by perseverance, work out a cure.

Returning from this digression, it remains for me to speak of the state of the burnt parts at and since the time of their cicatrization.

For several weeks the new cuticle, of the inner part of the left hand, was in a constant state of exfoliation and reproduction. At first it was separated by a thin stratum of serous fluid, and would come off in large coriaceous flakes, the operation being attended with considerable itching and soreness; but gradually the process of effusion ceased, and the scales became branny, in which form they continued to fall for more than a year. Their reproduction, indeed has not yet entirely ceased.

As the period of cicatrization approached, the granulations became affected with a distressing *neuralgia*, which was violent, for six or eight months, and has not yet, at the end of nearly two years entirely ceased. It would be difficult to characterize the different kinds of pain which the patient endured for the first half of this period. Sometimes it was dull and aching, but oftener acute and suddenly radiated from a point through the whole eschar. At other times, it resembled the sensation produced in the extremities of a nerve, by pressure or a blow on its trunk; again it was a sense of formication, and then of urtication; but more commonly the patient could only compare it with the imaginary effect of millions of fine, red hot needles, running in all directions through the new flesh. Its onset was frequently as sudden, and almost as transient, as an electric shock; suggesting to the mind of the patient, that it might be owing to the imperfect conducting power of the new nerves. Throughout the whole period, the parts were liable, at times, to a sense of violent spasmodic contraction, and in this condition, if pressed upon, felt almost as hard as cartilage. Several circumstances were observed to exert an influence on this affection.

1. When the patient was dyspeptic or otherwise indisposed, it was apt to be more violent.

2. When the parts were long exposed to the cold, it was made worse. Their calefacient power was, and remains greatly reduced. This is more remarkable in the middle finger of the right hand, than any other part. The ball only of the finger, was burnt; but under a moderate exposure to cold, the whole finger, quite up to the hand, looses its heat so rapidly, as to place it in contrast with the others. In this condition its extremity is generally neuralgic.

3. When the hands hung down, the neuralgia was suddenly and insupportably aggravated. For several months it appeared to the patient, upon letting his left hand fall by his side, that its continuance in that position would excite general convulsions—such were his feelings on the subject. If, however, he held a weight, or grasped any solid body, the distress was less poignant.

4. For many months, a kind of neuralgic flash or explosion, was the invariable consequence of a deep inspiration, whether made in sneezing, coughing, sighing, or as an experiment. Struck with this effect, the patient verified it by innumerable observations, and was not long in discovering (what seemed to render the fact still more curious) that the shock produced by a second deep inspiration, was much less, than that attendant on the first; and that upon continuing to respire, in that manner, a third or fourth time, the neuralgia would cease to be reproduced. After a few minutes, however, it could be re-excited by the same means, with its characteristic violence. I state the fact without attempting its explanation.

5. The effect of pressure on this neuralgia was highly beneficial, but the relief ceased with the compression. It was surprizing to the patient, to observe how much compression the recent granulations and eschar could sustain, without any unpleasant sensation—but the reverse. For months he was under the necessity of devising new modes of applying pressure, as the situation of the parts affected rendered every contrivance imperfect in its operation, and he was often obliged to seize and powerfully compress the principal eschar with the other hand so as to relieve the neuralgia when it became insupportable. For several weeks after cicatrization had commenced, he could not sleep at night, without placing the left hand under his pillow, in such a manner that the weight of his head would rest upon the eschar.

A great variety of applications were advised and employed for this formidable affection, but with little good. Of pressure I have just spoken. Its effects were but palliative. Covering the parts with varnished silk, so as to confine the perspiration, and exclude the air, was beneficial, and for more than a year it was found necessary to keep them protected with soft leather. The effusion of cold water over the parts affected was grateful, and often relieved the neuralgia, especially, when it was of an acrid and burning character, but its effects were transient. The tincture of opium, extract of belladonna and other narcotics afforded but little relief. Emollient poultices increased the irritation. Alcohol did good, and a solution of the nitrate of silver was found serviceable ; but like every other application, the relief it afforded was inconsiderable. Time only has been efficacious ; but even the lapse of nearly two years, has not entirely overcome the affection.

With respect to the specific sensibility of the new parts, something should be said. When they are passed over hard surfaces, the sensation excited in them is rather acute and sometimes painful, but in no degree specific or intelligible ; and hence the burnt fingers cannot be associated with the others in any operation that requires delicacy of touch. For a year or more, they could not feel the impulse of a soft and blunt body, such as the stroke of the artery at the wrist, though the patient could perceive, without the aid of his sight, that the affected finger was applied to the skin with the rest. But to the impress and the abstraction of heat, the new parts have always been morbidly sensible—suffering from both, much sooner than the corresponding sound parts. In this respect, indeed, the injured fingers bear to the uninjured, a relation similar to that which the *differential* thermometer of Leslie bears to the *mercurial* thermometer of Fahrenheit. The developing and resisting power of the new flesh, for caloric are, in fact, both reduced, while their sensibility to that agent is increased.

Cincinnati June, 1830.

SUPPLEMENT. As a supplement to the foregoing paper, we publish the following extract from Mr. Travers' valuable work on constitutional irritation under the impression that it is not in the hands of our readers generally.

" *Treatment of burns.*—Burns and scalds are unattended with hemorrhage. No species of injury exhibits the symptoms of prostration in a more marked degree than these, when of a severe description. The remedy upon which I rely in burns inducing this state, is brandy in gruel, in the proportion of one part of the former to two of the latter. This is administered in doses of a table-spoonful or two at short intervals, until we obtain a sufficient distinctness and fulness of the pulse at the wrist, with a corresponding improvement of complexion and temperature of the surface. The bowels should be simply kept unloaded by means of castor oil or common injections. If there be much stupor, and the bowels have been previously costive, I prefer to give ten or fifteen grains of the compound calomel and scammony powder. The use of opium, a remedy still generally employed on account of the patient's sufferings, I have abandoned, being convinced by experience, that in small doses it is inefficacious, and in large ones injurious. I have before observed, that pain is a good symptom in burns. I need scarcely observe that the exhibition of the stimulus requires unceasing and judicious attention ; it must be intermitted, if the pulse become bounding, and the patient flush. Nevertheless in deep and very extensive burns of adult subjects, I have frequently found it necessary to persevere in its use for a week or ten days at lengthening intervals ; occasionally intermitting, and always gradually withdrawing it. Not only has the partial remission of this plan been in some instances imperative, when re-action was permanently established, but symptoms of the head or chest have made their appearance which required blood-letting, either topical or general ; and a smart purge of calomel and scammony I have found on such occasions very useful. In deep burns the external remedy which I prefer is the turpentine and olive-oil liniment, or turpentine and cetaceous ointment, upon the principle, though not exactly the plan, recommended by Dr. Kentish of Newcastle, and subject, after suppuration commences, to such changes as the state of the surface may require. This appears to me to coincide advantageously with the internal use of the stimulant. The lime-water and milk liniment, and when suppuration has become abundant, the calamine or chalk powder lightly strewed over the whole surface, and defended by the same or simple ointment thinly spread on linen, is the local treatment which I adopt in superficial burns.

Upon the whole, the results of the stimulant treatment of burns, which I have more strictly pursued since my attention has been directed to the subject of this Essay, have proved highly satisfactory."

